# Haemato-biochemical, mutagenic, and histopathological changes in *Oreochromis niloticus* exposed to BTX

**DOI:** 10.1007/s11356-023-26604-2

**Published:** 2023-04-01

**Authors:** Alaa El-Din H. Sayed, Shaimaa K. Idriss, Sary Kh. Abdel-Ghaffar, Asmaa A. A. Hussein

**Affiliations:** 1 Molecular Biology Researches & Studies Institute, Assiut University, 71516 Assiut, Egypt; 2 Zoology Department, Faculty of Science, Assiut University, 71516 Assiut, Egypt; 3 Department of Fish Disease and Management, Faculty of Veterinary of Medicine, Assiut University, 71516 Assiut, Egypt

**Keywords:** Acridine orange, Erythrocytes, DNA damage, Fish, BTX

## Abstract

The study of the DNA damage response in erythrocytes after exposure to volatile organic compounds (VOCs) can present evidence for its potential effect as genotoxic- biomarkers for environmental pollution. Although VOCs are dangerous pollutants, still little is known about hemotoxic, cytotoxic, and genotoxic effects of such pollutants on fish. We optimized an assay method for apoptosis and DNA damage in erythrocytes of adult tilapia fish after 15 days exposure to benzene (0.762 ng/L), toluene (26.614 ng/L), and xylene (89.403 ng/L). The highest level of apoptosis and DNA damage were recorded in benzene-exposed fish, as was the highest level of histopathological alterations in gills, liver, and kidney. The imbalance of the antioxidants profile explained the stress-case reported in exposed fish. These results suggest that hemotoxic, cytotoxic, genotoxic, and tissue damage were recorded after exposure to BTX in *Oreochromis niloticus*.

## Introduction

Although volatile organic compounds (VOCs) are dangerous pollutants that are present in many environments, little is known about their hemotoxic effects on aquatic organisms. Solvents, paints, glues, and other products used and stored at home and at work can release these chemicals when fuels, such as gasoline, wood, coal, or natural gas, burn. Ground-level ozone, or smog, is formed when VOCs react with nitrogen oxides. There are many types of VOCs, including formaldehyde, toluene, chloroform, gasoline, benzene, formaldehyde, toluene, chloroform, 7,12-dimethylbenz[a]anthracene- (DMBA), and xylene (Dakrory et al. 2015, U.S. National Library of Medicine 2012).

Benzene, toluene and xylene are volatile organic solvents used widely in industry, they are known for their toxic effect on liver, kidney, blood and brain (Snyder & Hedil 1996, Wong 1995). Studies on intraperitoneal administration of benzene, toluene and xylene have been performed (Anderson & Richardson 1981).

Benzene is an important environmental pollutant compound, and due to its lipid soluble nature, it tends to accumulate in tissues. It is absorbed into the liver and oxidized once inhaled, consumed or applied to the skin, while the remaining molecules are metabolized through the cytochrome P450 (CYP) system in the liver, bone marrow and other tissues (Kumar & Singh 2003). In addition to being an ingredient in motor fuels, it is also used as a solvent in oils, resins, resins, inks, paints, plastics, and rubber. It is also used in the extraction of oils from seeds and nuts. Benzene toxicity may be caused by reactive oxygen species (ROS) production and a binding to cellular macromolecules that cause damage. This solvent produces ROS which causes damage to cellular macromolecules (Fahmy & Mohamed 2015, Parke 1996). They play a role in activating gene expression, cell proliferation, and cell death, as well as enhancing lipid peroxidation (Holmstrom & Finkel 2014). A benzene ring is attached to a methyl group, which is a colorless industrial hydrocarbon. The world's household products as well as the rubber and petroleum industries use toluene as a solvent (Niaz et al. 2015). Furthermore, xylene serves as a solvent in medicine and industry. Petrol, coal, and wood tar naturally contain this solvent as colorless, sweet-smelling gases or liquids. Three forms of xylene exist: ortho, meta, and para (Kandyala et al. 2010). Rubber, printing, and leather industries use xylene extensively as a solvent. Paints, cleaning agents, and varnishes can also be thinned with it. Airplane fuel and gasoline contain small amounts of xylene (Rajan & Malathi 2014).

As fish are highly sensitive to pollutants, hemo-biochemical parameters, antioxidants, and mutagenic biomarkers are good indicators for the assessment of aquatic pollutants, oxidative stress caused by pollutants, and evaluating the environmental health (Ahmad et al. 2000, Almeida et al. 2002, 2012, Martínez-Álvarez et al. 2005, Sayed et al. 2016).

The assessment of pollution's impact on biomonitoring is based on the evaluation of histopathological alterations, which are key tools and sensitive indicators in the field of environmental toxicology (Li et al. 2015a). Khaled et al., (2022) has been used the histopathological and molecular changes in liver, kidney and gills of *Oreochromis niloticus* to assess the effects of an environmentally concentration of benzene, toluene, ethylbenzene and xylene (BTEX).

Nile tilapia, *Oreochromis niloticus* are a widely cultivated freshwater fish species because of their ability to withstand environmental changes and stresses. The species is widely used as a model species in ecotoxicological studies, so it was selected because of its importance in aquaculture (Hamed et al. 2019). This study aims to investigate the effects of different doses of benzene, toluene, and xylene on the hematological, biochemical, antioxidant, apoptosis, and histopathology of liver, kidney and gills tissues of *Oreochromis niloticus*.

## Materials and methods

### Fish

Nile Tilapia (*Oreochromis niloticus*) (weight 100–150 g; length 10–15 cm) were used in this experiment. The fish were parasitic-free according to American Fisheries Society, Fish Health Section (AFS-FHS 2017). Fish were acclimated in glass tanks (100 cm × 70 cm × 50 cm) under physicochemical conditions of the rearing water as: conductivity 260.8 mMcm^−1^; pH 7.4; dissolved oxygen 6.9 mg L^−1^; temperature 20.5℃; photoperiod 12:12 hlight:dark). During the acclimatization period, fish were fed commercial feed (30% protein) about 3% of their body weight. 50% of the water was changed daily with re-dosing.

### Experimental design

The Nile Tilapia (*Oreochromis niloticus*) was randomly distributed after adaptation period into four groups. Each group consists of 30 samples (each replicate 10 fish) and was kept in glass containers having the same volume of water (100 L). The animals in each group were treated with water mixed with BTX:

- **○ Group Ӏ:** The animals were fed on the control diet**.**
- **○ Group ӀӀ:** The animals were exposed to benzene (0.762 ng/L) for 15 days.
- **○ Group ӀӀӀ:** The animals were exposed to toluene (26.614 ng/L) for 15 days.
- **○ Group ӀV:** The animals were exposed to xylene (89.403 ng/L) for 15 days.

The concentration of the tested chemicals was environmentally relevant and selected in accordance with the method of Kasemy et al. (2019).

A random sample of six fish from each group were selected after 15 days, and ice anesthesia was administered to reduce stress during processing (Hamed et al. 2019), and for further analysis of hemato-biochemical parameters, antioxidant enzymes, and erythron profiles, blood was collected from the caudal vein.

### Hemato-biochemical parameters

Various hematological indices (red blood cells [RBC's] and white blood cells [WBC's] count; Differential WBC's; blood Platelets; Haematocrit level [Hct], Hemoglobin level [Hb]; Erythrocyte indices including mean corpuscular hemoglobin [MCH], Mean corpuscular volume [MCV], and mean corpuscular hemoglobin concentration [MCHC]), were determined according to Mekkawy et al. (2011).

Blood samples for biochemical parameters were collected from fish without anticoagulant agent for serum collection. Colorimetric determinations of the different important biochemical indices: alanine aminotransferase (ALT), alkaline phosphatase (ALP), aspartate aminotransferase (AST), cholesterol, creatinine, uric acid, albumin, globulin, glucose, and total protein were performed according to Hamed et al. (2019) by using spectrophotometer in a wavelength ranging from 340 to 546 nm based on the kits manufacturers of each target parameters.

### Oxidative stress biomarkers

Antioxidants kits from Biodiagnostic Company, Cairo, Egypt were used to estimate superoxide dismutase (SOD), catalase (CAT), total antioxidant capacity (TAC), and total peroxide (TPX) according to the procedure of (Aebi 1984; Harma et al. 2005; Koracevic et al. 2001; Nishikimi et al. 1972). Malondialdehyde (MDA) was measured using a thiobarbituric acid reaction (Ohkawa et al. 1979).

### Mutagenic biomarkers

Cell viability assay was conducted using a hemocytometer and the trypan blue dye exclusion test. These tests used only samples with a minimum cell count of 10^6^ cells/100 µl and a viability of > 90%.

In order to detect apoptosis using acridine orange (AO) stain (Cat. No. A1031, Life Technologies, Carlsbad, CA, USA) in red blood cells (RBCs), a modified protocol (Sayed et al. 2016) was used. Blood smears were washed in 1 × PBS (pH = 7.2), then added AO buffer (17 µg/l AO in 1 × PBS buffer) 30 min in the dark. Wash the slides every 30 min with 1 × PBS four times, then fixation in paraformaldehyde 4% for 5 min.

A neutral comet assay was used as described (Hidaka et al. 2009) with minor modifications (Sayed et al. 2017). 2 -8 × 10^4^ red blood cells/µl PBS (pH = 7.2) were mixed with 300 µl of 1% low-melting temperature agarose (Lonza) in PBS, then the mixture layered on a glass microscope slide previously coated with 1% agarose, then covered with coverslip. A fresh lysis buffer (2% SDS and 30 mM EDTA) was prepared, and the slide immersed in it for 30 min after it was placed on ice for 3 min to gel the agarose. Afterwards, TBE was used to clean the slide, and electrophoresis at 20 V for 25 min was conducted. A solution of propidium iodide at 100 ng/ml was soaked on the slide for five minutes. A 14 MP OMAX camera (A35140U3) was used to observe cells under a fluorescence microscope (BX-50, Olympus). Based on the comet image of each cell, CASP software calculated tail moment scores (Końca et al. 2003).

### Histopathology examination

After 15 days, 3 fish from each group (control and treated) were manipulated; tissue samples (gills, liver and kidney) were dissected anatomically from each fish and washed by neutral saline. Each specimen of tissue was dehydrated by 70, 90, 96, and 100% alcohol after fixing in neutral buffered formaldehyde, after that cleared with methyl benzoate and embedded in paraffin wax. Cut thin sections of 5 microns thickness. After that dewaxed slide and rehydrated, then stained H & E (Feldman & Wolfe 2014). All sections were histopathologicaly examined and photographed using an Olympus CH30 microscope. The score of pathological lesions was as: (-), absent; ( +), minimal; (+ +), moderate; (+ + +), severe; a maximum score (+ +  + +) (Meydan et al. 2019).

### Statistical analysis

The homogeneity of variance was assumed for raw data. Moreover, in the absence of interactions, the pattern of variations was recorded by one-way ANOVA considering Tukey-HSD test for multiple comparisons using IBM-SPSS package version 21 (IBM-SPSS, 2012) at 0.05 significance.

## Results

### Haemato-biochemical parameters

The values of hematological parameters of control and 15-days exposed *O. niloticus* are given in Table 1.

**Table 1:** The basic data (N = 4) of hematological parameters of *O. niloticus* exposed to Benzene, Toluene and Xylene for 15-day exposure

Parameters	RBC million/mm3	Hb g/dl	Hct %	MCV µm^3^	MCH Pg	MCHC %	Platlates Thousands/ mm3	WBC Thousands/ mm3	Lymphocyte %	Monocytes %	Neutrophils %	Eosinophils %
Control	1.95 ± 0.01 (1.91–1.97)^c^	8.35 ± 0.06 (8.2–8.5)^c^	25.95 ± 0.47 (24.9–27)^b^	133.24 ± 1.96 (130.1–138.46)^a^	42.88 ± 1.96 (42.35–43.59)^a^	32.2 ± 0.33 (31.48–32.93)^a^	315 ± 0.56 (314–316)^b^	846.75 ± 3.52(841–857)^b^	88 ± 0.71 (86–89)^ab^	2.75 ± 0.25 (2–3)^ab^	7 ± 0.41 (6–8)^a^	2.25 ± 0.5 (2–3)^c^
Benzene	1.49 ± 0.03 (1.42–1.55)^a^	7.62 ± 0.05 (7.5–7.7)^a^	23.13 ± 0.34 (22.2–23.8)^a^	299.25 ± 2.33 (149.03–160.27)^c^	51.16 ± 1.11 (49.35–54.23)^bc^	33.00 ± 0.64 (31.93–34.68)^a^	299.25 ± 3.82(289–306)^a^	776 ± 28.00 (727–825)^a^	90 ± 0.41 (89–91)^c^	2 ± 0 (2–2)^a^	7.25 ± 0.49 (6–8)^a^	0.75 ± 0.5 (0–1)^a^
Toulene	1.54 ± 0.01 (1.52–1.55)^a^	7.93 ± 0.06(7.8–8.1)^b^	23.78 ± 0.37 (23.1–24.8)^a^	146.08 ± 2.14 (140.85–151.22)^b^	48.7 ± 2.14 (47.56–50)^b^	33.36 ± 0.64 (31.45–34.2)^a^	303.5 ± 4.86(289–309)^ab^	836.75 ± 0.75 (835–838)^b^	86.75 ± 0.48 (86–88)^a^	3.5 ± 0.29 (3–4)^b^	7.75 ± 0.25(7–8)^a^	2 ± 0 (2–2)^bc^
Xyelen	1.63 ± 0.01(1.61–1.64)^b^	7.975 ± 0.08 (7.8–8.1)^b^	24.3 ± 0.45 (23.3–25.5)^a^	158.03 ± 2.38 (153.29–164.52)^c^	51.88 ± 2.38 (50.32–53.29)^c^	32.85 ± 0.66 (31.76–34.76)^a^	310.5 ± 0.5 (310–312)^ab^	832.25 ± 4.77 (824–845)^b^	89.75 ± 0.25 (89–90)^bc^	2.5 ± 0.29 (2–3)^a^	6.5 ± 0.29 (6–7)^a^	1.25 ± 0.5 (1–2)^ab^

Different letters indicate significance at P < 0.05

Benzene was found to significant variations (P < 0.05) in all hematological parameters (RBC, Hb, Hct, MCV, MCH, platelets, WBC, lymphocyte, monocyte and eosinophils) except in MCHC and neutrophils compare with control group. Whereas toluene was found to significant variations (P < 0.05) in RBC, Hb, Hct, MCV, MCH, platelets and monocyte only. Xylene was found to significant variations (P < 0.05) in RBC, Hb, Hct, MCV, MCH, lymphocyte and eosinophils only compare with control group. Finally, the effect of benzene is more than toluene and xylene effects in hematological parameters.

The values of biochemicals parameters of control and 15-days exposed fish; *O. niloticus* is given in Table 2.

**Table 2:** The basic data (N = 4) of biochemical parameters of *O.niloticus* exposed to Benzene, Toluene, and Xylene for 15-day exposure

Parameters	AST u/l	ALT u/l	ALP u/l	Glucose mg/dl	Total Protein g/dl	Creatinine mg/dl	Albumin g/dl	Globulin g/dl	AG
Control	54.82 ± 0.56 (53.8–55.9)^a^	28.65 ± 0.22 (28.2–29.2)^a^	25.03 ± 0.26 (24.7–25.8)^a^	102.5 ± 1.24 (99.8–105.8)^a^	4.4 ± 0.07 (4.2–4.5)^a^	0.57 ± 0.03 (0.54–0.64)^a^	1.12 ± 0.03 (1.06–1.21)^a^	2.13 ± 0.01 (2.12–2.14)^a^	0.53 ± 0.016 (0.5–0.57)^a^
Benzene	63.68 ± 0.40 (62.8–64.7)^b^	30.98 ± 0.36 (29.9–31.4)^b^	28.65 ± 0.51(27.8–29.8)^b^	113.68 ± 2.21 (108.7–119.2)^b^	5.95 ± 0.25 (5.2–6.3)^b^	0.75 ± 0.01 (0.74–0.78)^b^	1.53 ± 0.013 (1.49–1.55)^b^	2.36 ± 0.03 (2.32–2.45)^b^	0.65 ± 0.01 (0.61–0.67)^b^
Toluene	53.93 ± 1.01 (51.7–56.6)^a^	27.93 ± 0.44 (26.8–28.8)^a^	26.28 ± 0.28 (25.8–26.9)^ab^	110.91 ± 1.86 (105.36–113.2)^b^	4.93 ± 0.28(4.1–5.3)^a^	0.59 ± 0.032 (0.52–0.66)^a^	1.23 ± 0.05(1.14–1.37)^a^	2.16 ± 0.01(2.14–2.19)^a^	0.56 ± 0.03(0.52–0.64)^a^
Xylene	56.3 ± 0.29 (55.7–56.8)^a^	30.8 ± 0.45(29.5–31.5)^b^	28.75 ± 1.35(26.6–32.4)^b^	109.1 ± 1.75(104.2–112.5)^b^	5.125 ± 0.13(4.8–5.4)^a^	0.64 ± 0.025(0.57–0.68)^a^	1.21 ± 0.03(1.11–1.26)^a^	2.22 ± 0.03(2.14–2.28)^a^	0.55 ± 0.01(0.52–0.57)^a^

Different letters indicate significance at P < 0.05

The effect of benzene was found to highly significant increased (P < 0.0001) in all biochemical parameters (AST, ALT, ALP, glucose, total protein, creatinine, albumin, globulin and A/G ratio) compare with control group. However, there is no significant effect of Toluene in biochemical parameters except for glucose and albumin only. Whereas Xylene was found to significant increased (P < 0.05) in all biochemical parameters except for AST, albumin and A/G ratio. The effects of benzene and xylene are more than toluene effect in biochemical parameters.

### Antioxidants alterations

The values of antioxidant of control and 15-days exposed *O. niloticus* are given in Table 3.

**Table 3:** The basic data (N = 4) of antioxidant and apoptosis percentage of *O. niloticus* exposed to Benzene, Toluene, and Xylene for 15-day exposure

Parameters	MDA (nmol/ml)	TAC (Mμ/L)	TPX (Mμ/L)	OSI%	SOD (IU/L)	CAT (IU/L)	Apoptosis %
Control	4.14 ± 0.01(4.12–4.15)^a^	1.06 ± 0.00(1.05–1.06)^a^	1.68 ± 0.03(1.65–1.76)^a^	159.16 ± 2.81(155.08–167.3)^a^	11.84 ± 0.03(11.78–11.9)^a^	10.89 ± 0.02(10.85–10.94)^a^	2.38 ± 0.15(0–5)^a^
Benzene	4.43 ± 0.01(4.42–4.45)^c^	1.10 ± 0.01(1.09–1.12)^c^	1.92 ± 0.03(1.86–1.98)^b^	174.64 ± 2.18(170.33–180.16)^b^	12.55 ± 0.05(12.42–12.65)^b^	11.46 ± 0.02(11.42–11.52)^b^	13.5 ± 0.42(7–22) ^d^
Toulene	4.22 ± 0.01(4.21–4.23)^b^	1.074 ± 0.00(1.07–1.08)^b^	1.73 ± 0.05 (1.65–1.86)^a^	160.82 ± 4.70(153.06–172.7)^a^	12.32 ± 0.03 (12.24–12.35)^b^	11.34 ± 0.04(11.24–11.44)^b^	7.53 ± 0.33(1–15)^c^
Xylene	4.24 ± 0.01(4.22–4.25)^b^	1.08 ± 0.00(1.08–1.09)^b^	1.77 ± 0.01(1.76–1.78)^a^	163.21 ± 0.87(161.76–165.43)^a^	12.32 ± 0.14(11.9–12.5)^b^	11.34 ± 0.05(11.2–11.42)^b^	4.62 ± 0.19(3–11)^b^

MDA; monoaldehyde, TAC; total antioxidant capacity, TPX, total peroxides, OSI; oxidative stress index, SOD; superoxide dismutase, and CAT; catalase. Different letters indicate significance at P < 0.05

Benzene was found to highly significant increased (P < 0.0001) in all antioxidants (MDA; monoaldehyde, TAC; total antioxidant capacity, TPX, total peroxides, OSI; oxidative stress index, SOD; superoxide dismutase, and CAT; catalase) compare with control group. Toluene and Xylene was found to significant increased (P < 0.05) in all antioxidants except for TPX and OSI only compare with control group. The effect of benzene is highly significant effect compared to toluene and xylene.

### Mutagenic changes

**Fig. 1:**
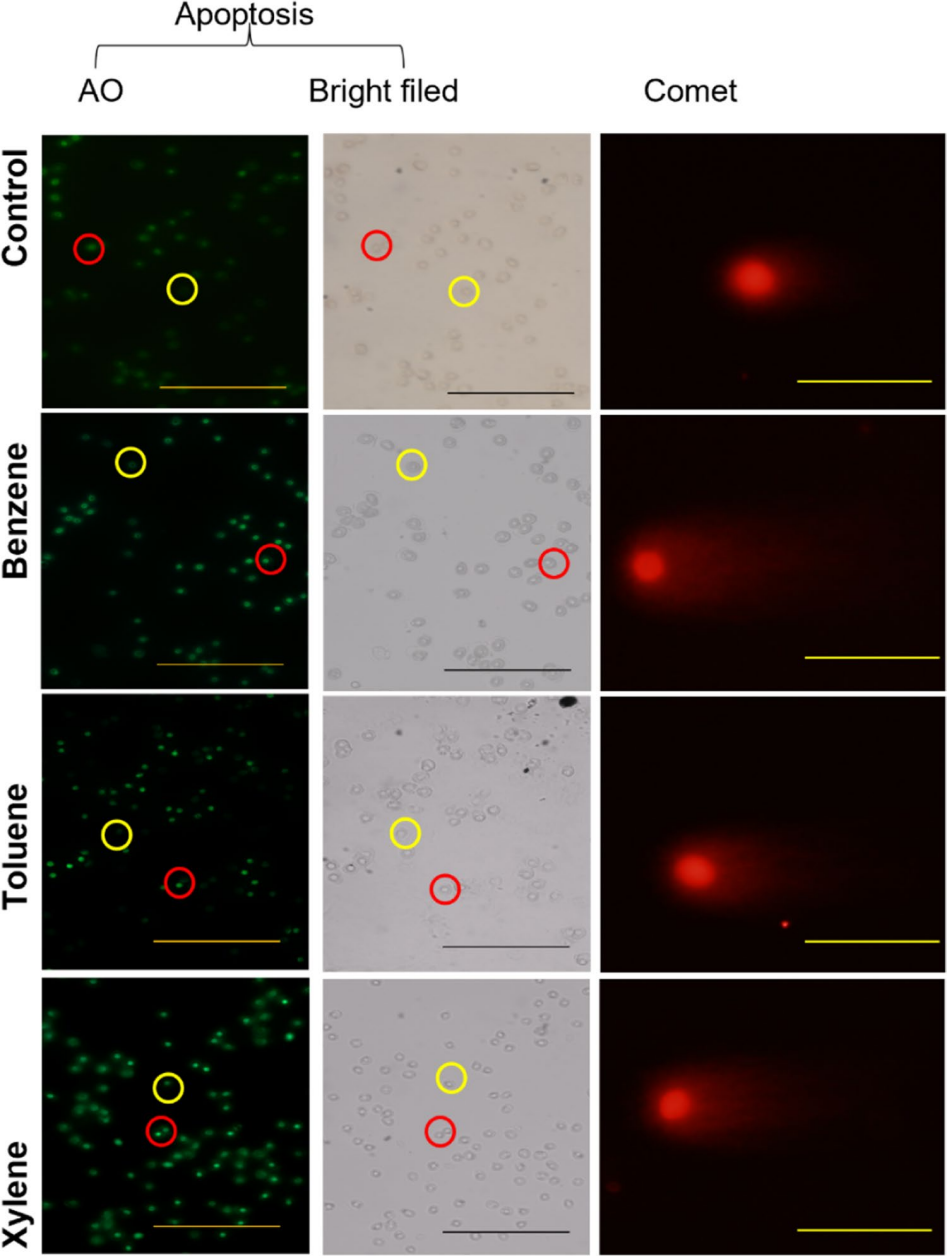
Apoptosis detection and DNA damage in erythrocytes Nile tilapia exposed to BTX for 7 days. Cells appeared light green were apoptotic. Cells stained with ethidium bromide were considered to have DNA damage. (Scale bar = 50 μm)

**Fig. 2:**
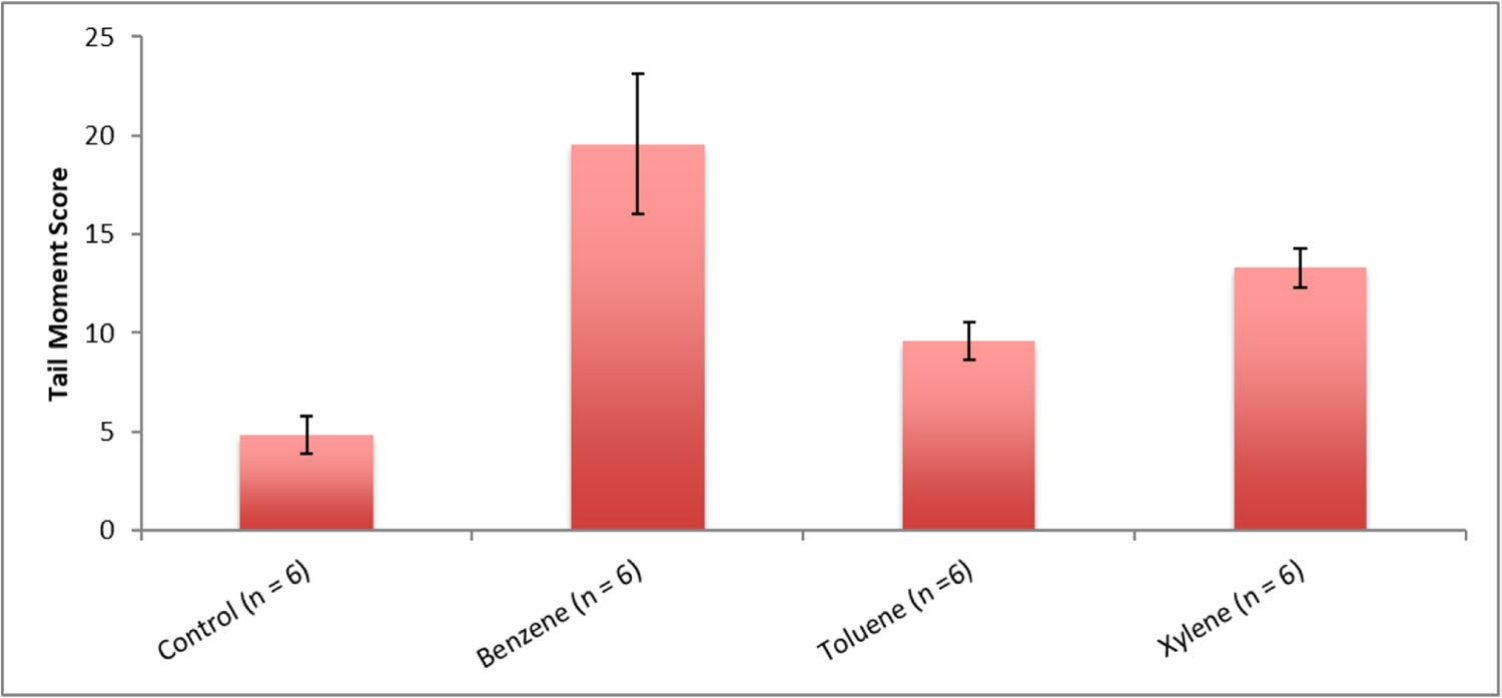
The mean tail moment score in erythrocytes of Nile tilapia exposed to BTX

The values of apoptosis percentage of control and 15-days exposed *O. niloticus* are given in Table 3 and Figs. 1 and 2.

Exposure of *O. niloticus* to benzene, toluene, and xylene induced DNA damage in terms of tail moment depending on the concentrations of benzene, toluene and xylene (Table 3). According to one-way ANOVA, benzene affect significantly (P < 0.0001) the values of the mean tail moment score (Table 3 and Figs. 1 and 2) in comparison to toluene and xylene. On the other hand, benzene, toluene and xylene was found to highly significant increased (P < 0.0001) in apoptosis compare with control group.

Figure 2 shows the comet assay after BTX exposure. All groups of fish had significantly different tail moments based on statistical analysis of their data (Fig. 2). The tail comet score of exposed fish samples increased significantly compared to their respective controls. Toluene and xylene caused the greatest DNA damage to exposed fish, while benzene caused the least DNA damage.

### Histopathological changes

**Fig. 3:**
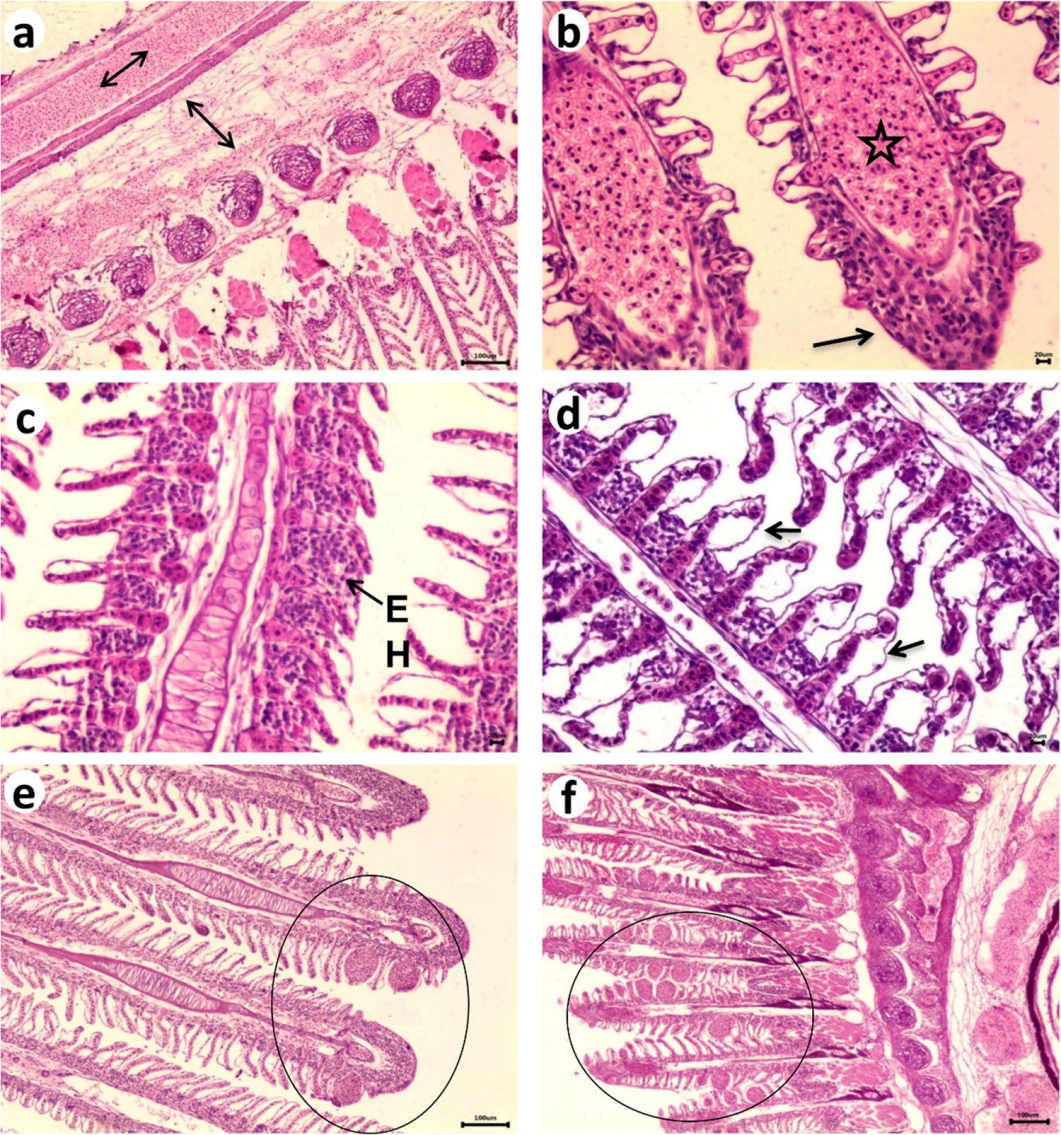
Photomicrograph of Nile tilapia gills, H&E-stained Showing, a- edema and congestion of gill arch (double arrow), b- Dilatation and congestion of blood vessels of primary lamellae (star) and secondary lamellae, fusion on the tips of gill filament (arrow) C- epithelial hyperplasia (EH) of primary lamellae which lead to fusion of secondary lamellae d- epithelial detachment (lifting)of the secondary gill lamellae (arrow). e, f- lamellar telangictasis (circle)

Normal histological structure was observed in gills, liver and kidney sections of the control Nile tilapia. The exposure to 3 treatments (benzene, toluene, and xylene) resulted in edema and congestion of gill arch in all experimental fish Fig. 3a. Dilatation of blood vessels of primary and secondary lamellae with congestion which appeared obviously after toluene exposure Fig. 3b. Thickening of the primary and secondary gill lamellae due to epithelial hyperplasia which lead to fusion on the tips of gill filament in all exposed fish Fig. 3c, but after xylene it present in a focal manner. Necrosis and detachment of lamellar epithelium were clear in all experimental fishes Fig. 3d. Lamellar telangictasia observed in a sever manner after toluene and xylene exposure Fig. 3e, f.

**Fig. 4:**
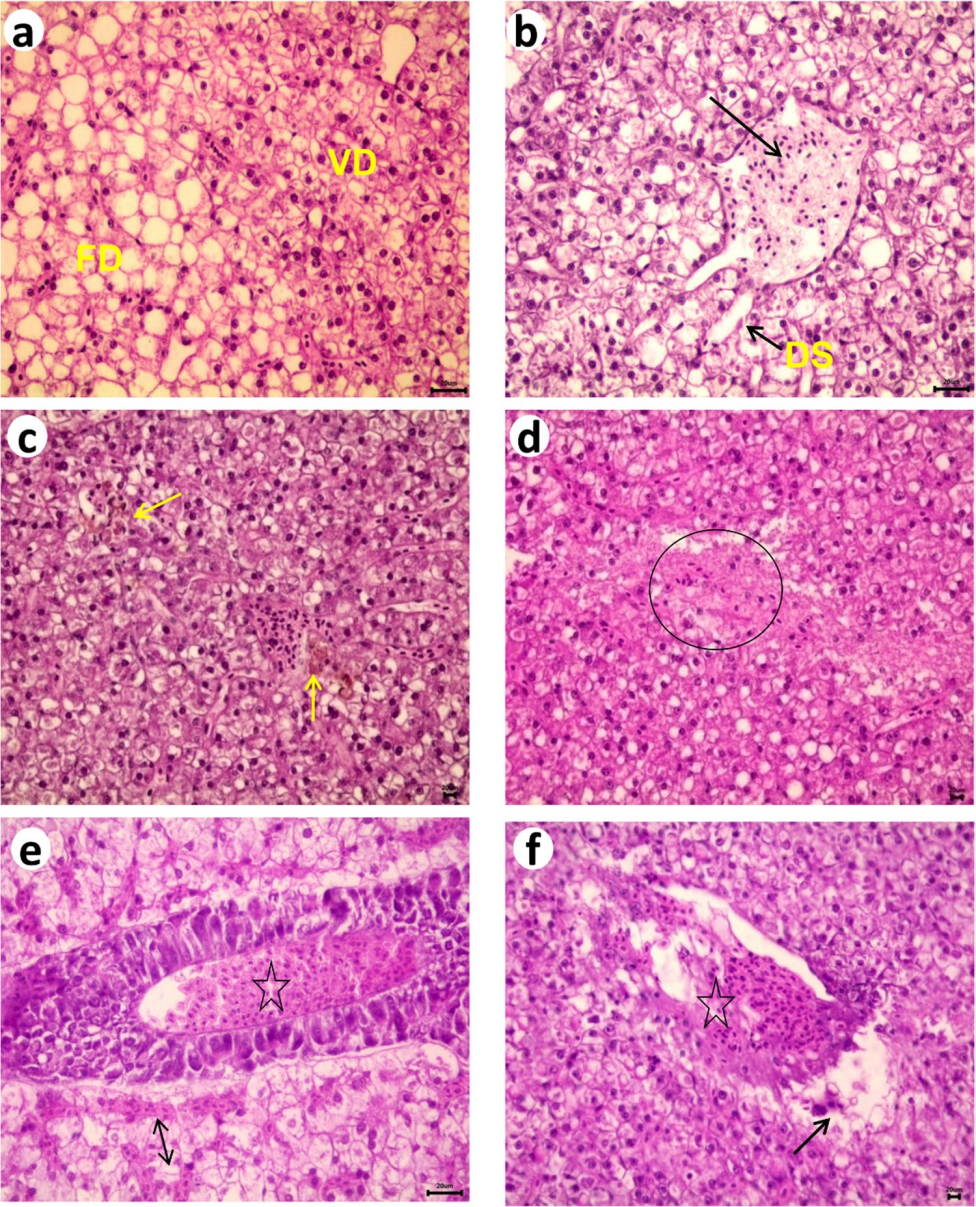
Photomicrograph of Nile tilapia liver, H&E-stained Showing, a- vacuolar degeneration (VD) with fat degeneration (FD), b- congested central vein (arrow) and dilated sinusoids (DS) after toluene exposure. After benzene exposure, c- Melanomacrophages aggregation (arrow) and d- Hepatocellular necrosis (circle). After xylene exposure, e- Congestion of hepatopancrease (star) and sinusoids (arrow) and f- Thrombus inside central vein (star)

Liver tissues of the Nile tilapia exposed to concentrations of benzene, toluene and xylene displayed sever dissociation of hepatocytes. Vacuolar degeneration of hepatocytes was recorded in all treated fish to a low degree but noticed obviously in fish treated with xylene. Steatosis (Fatty degeneration) was observed in a zonal and diffuse manner after toluene exposure Fig. 4a. Congestion and of central vein were recorded in all exposed fish. Severity of sinusoidal dilatation observed in a different degree mild, moderate and severe respectively Fig. 4b. Mild aggregation of melanomacrophages Fig. 4c. The focal area of coagulative necrosis of hepatocytes was observed in all treated fish and become more severe and multifocal after xylene exposure Fig. 4d. Congestion of hepatopancrease was noticed in all treated fish but severity increased after xylene exposure Fig. 4e. Thrombosis was observed in hepatopancrease, portal veins and in central vein after xylene exposure and become prominent after toluene exposure Fig. 4f.

**Fig. 5:**
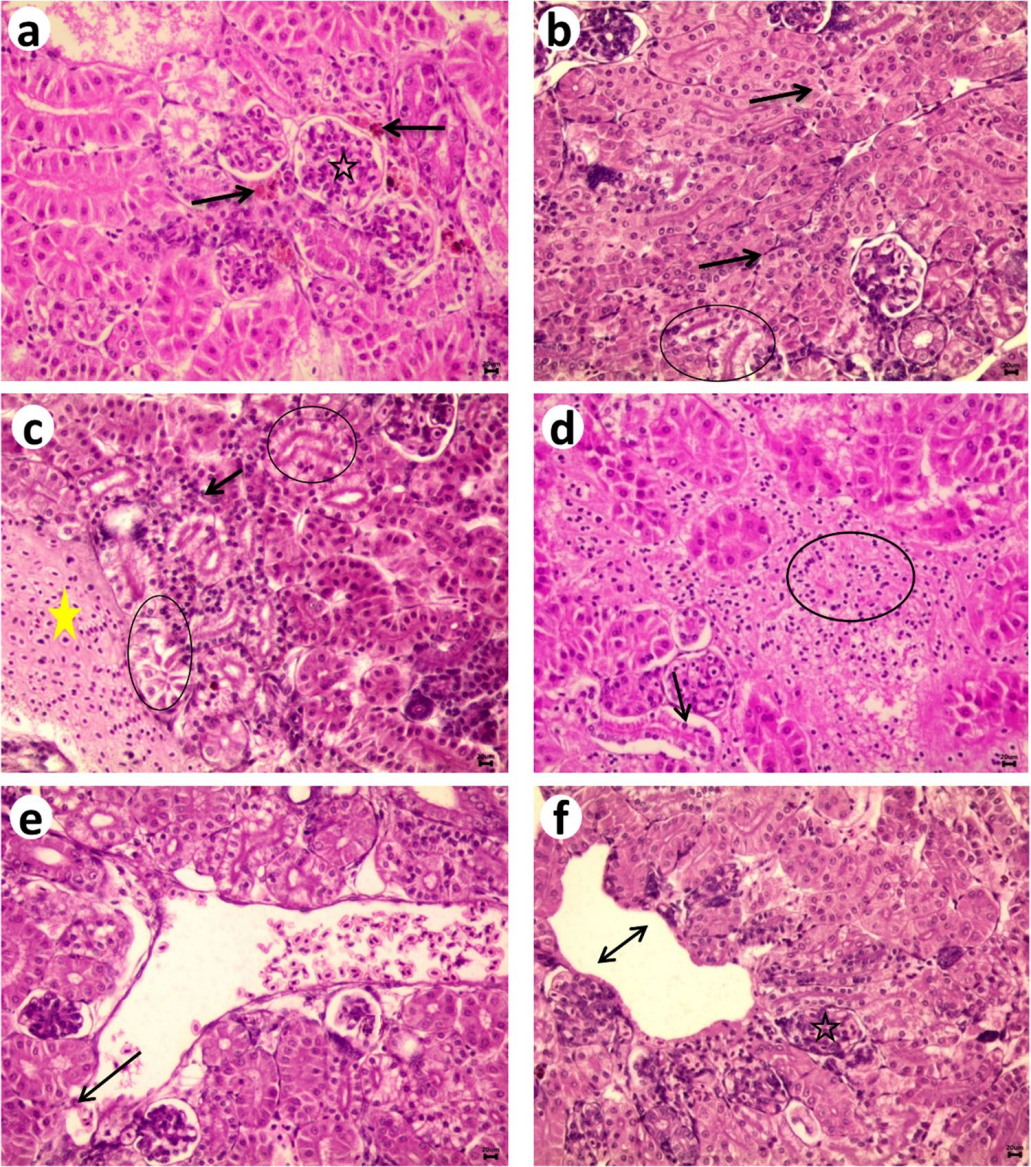
Photomicrograph of Nile tilapia kidney, H&E-stained Showing, a- melanomacrophages aggregation (arrow) with glomerular swelling (star). b, c- interstitial inflammatory cell infiltration (arrow), necrosis and vacuolar degeneration of renal tubular epithelium (circle), congestion of blood vessels (yellow star) d- diffuse Interstitial hemorrhages (circle) Separation of tubular epithelium from basement (arrow). e- necrosis of blood vessels wall (arrow). f- degeneration of blood vessels wall (double arrow) hypercellularity of glomeruli (star)

Renal damage also observed as moderate glomerular swelling with sever aggregation of melanomacrophages after xylene and toluene experiment Fig. 5a. Diffuse interstitial inflammatory cell infiltration Fig. 5b while, necrosis and vacuolar degeneration in epithelial cells lining renal tubules were noticed in all experimental fish Fig. 5c. Interstitial hemorrhages were observed Fig. 5d. Sever degeneration in the blood wall was cleared in all experimental fish Fig. 5e associated with hypercellularity of glomeruli Fig. (5f). The score of the pathological lesions were reported in Table 4.

**Table 4:** Effect of Benzene, Toluene, and Xylene on gills, liver, and kidney of *Oreochromis niloticus* exposed to BTX

Gills			
Lesions	Benzene	Toluene	Xylene
Gill arch: Edema of capillaries	+ + +	+ +	+ + +
Hemorrhage	+ + + +	+ + + +	+ + + +
Inflammatory cell infiltrations	+ + + +	+ +	+ + +
Gill filament (primary lamellae): Lamellar congestion	+ +	+ + + +	+
Dilatation of blood vessels	-	+ + + +	+ +
Epithelial hyperplasia	+ +	+ + +	+ + +
Epithelial necrosis	+ +	+ + + +	+ + +
Gill lamellae (secondary lamellae): Lamellar congestion	+ + +	+ + + +	+ + +
Lamellar telangictasis	-	+ + + +	+ + +
Lifting of epithelium (epithelial detachment)	+ + +	+ + + +	+ + + +
Curling of lamellae	+ + + +	+ +	+ +
Epithelial necrosis	+ + +	+	+ + + +
Fusion of adjacent lamellae	+ + +	+ +	+ + +
Liver			
Hepatic cord disarray	+ + +	+ + +	+ + +
Vacuolar degeneration of hepatocytes	+ + +	+ + +	+ + + +
Fatty degeneration of hepatocytes (steatosis)	+	+ + +	+ + + +
Sinusoidal dilatation	+ +	+ + +	-
Central vein dilatation	-	+ + + +	+ + +
Necrosis of hepatocytes	+ + +	+ + +	+ + + +
Congestion and necrosis of central vein	+	+ + +	+ + + +
Inflammatory cell infiltration in portal area	-	+	+ +
Hemorrhages within hepatocytes	+	-	+ + +
Degeneration and necrosis of sinusoids	-	+ +	+
Congestion of sinusoids	-	-	+ + +
Melanomacrophage aggregation	+ +	-	+
Congestion of hepatopancrease	+	+ + +	+ + +
Thrombosis	-	+ + +	+ +
Kidney			
Glomerular swelling	-	+ + +	-
Dilatation of bowman's space	+ +	+	-
Atrophy of glomerular tuft	+ +	-	-
Lymphocytic infiltration	-	+ +	+ + +
Cloudy swelling of tubular epithelium	+ +	+ + +	+ +
Coagulative necrosis of tubular epithelium	+ +	+ +	+ + +
Vacuolar degeneration of tubular epithelium	+ +	+ + +	+ + + +
Dilatation of tubular lumen with hyaline cast	+	+	+ +
interstitial inflammatory cell infiltration	+	+ + +	+ + + +
Accumulation of melanomacrophages	-	+ +	+ + + +
Interstitial Hemorrhage	-	+ + +	+ + + +
Edema of Blood vesseles	+ + +	+ + +	+ + +
Inflammatory cell infiltration	+ +	+ +	+ + +
Degeneration of blood vessels wall	+ + +	+ + + +	+
Perivascular hemorrhages	-	+ +	+ + +

## Discussion

In the routine clinical evaluation of fish exposed to toxicants, haematological indices are often associated with health status (Odioko & Daniel 2016). It is possible that fish respond to stress in a non-specific way, allowing them to cope with the condition and maintain their homeostasis (Adewoye 2010). The fish's health and wellbeing may be endangered if the stress is severe and persistent (Audu et al. 2014).

There was noticeable adverse impact on hematological and biochemical changes in the fish after exposure to organic solvents (Benzene, toluene, and xylene (BTX). There was significant decrease were observed in RBCs, Hb, Hct, Platelets, WBCs, monocyte, eosinophils and significant increase in MCV, MCH, and lymphocyte of the *O. niloticus* exposed to the various concentrations of organic solvent during the study. MCHC and neutrophil counts didn’t differ significantly between the groups.

A variety of toxicants were exposed to fish and similar results were reported (Ogamba et al. 2014). The observed variations in the haematological parameters of blood cells may because anemia and leucopenia as reported by (Luskova et al. 2002). These results were in agreement with Chris et al. (2022), who observed decreased in RBCs, HB, Hct, Platelets and MCHC and increased in MCV and WBCs in *Clarias gariepinus* when exposed to different doses of xylene.

Also, Ibrahim et al. (2011), who observes decreased Hb and Ht values when rat exposed to benzene. A rat exposed to xylene and benzene had reduced erythrocyte counts, hematocrits, and hemoglobin, as reported by d'Azevedo et al. (1996). Researchers found the same results in rats exposed to nitrobenzene in 1994 by Shimo et al. (1994). Benzene treatment also reduced haemoglobin concentration in rats, according to Escorcia et al. (1997). In some studies, reductants are believed to mobilize iron from storage proteins within the cells to explain this decrease (Minotti 1992; Shaw et al. 1988). CYP P450 and other hemeproteins may be synthesized with this released iron (Minotti 1992). According to this study, RBCs, Hb and platelets were decreased in albino rats exposed to toluene, in agreement with that of AI-Sahhaf and Sarhan (2016).

An investigation of biochemical and hematological changes during shoe making was conducted by Khan et al. (2013). In the study, platelet and neutrophil counts were significantly low, and blood glucose and hemoglobin levels were normal. However, hematocrit and mean corpuscle volume were low, as well as mean cell hemoglobin level and concentration. Compared to this, leukocytes, lymphocytes, eosinophils, and monocytes increased significantly. These results agree with Morcos et al. (2015) who observed decrease in hematological parameters (RBCs, HB, Platelets, monocyte and eosinophils) and increase in WBCs and lymphocytes in mice treated with xylene. Also, Saleh et al. (2007) who observed decline in hemoglobin and hematocrit value of albino rats exposed to 0.5 ml/kg body weight benzene. The effects of benzene on hematotoxicity have been studied extensively (Ahmad et al. 1994; d’Azevedo et al. 1996; Escorcia et al. 1997; Qu et al. 2002).

The changes observed in fish Hct can be attributed to the swimming frequency resulting from the increased concentration level of the toxicant, significant decline in the RBC, Hct, and hemoglobin due to the increasing doses of xylene (Chris et al. 2022). This agrees with Valores, (2005) who reported a low level of Hct and hemoglobin in an aquatic organism with sedentary behavior in some lentic water bodies and a higher value in active aquatic lives with spleen contraction which also resulted in a distorted shape and size of RBC as well (Dal'Bó et al. 2015; Gallo et al. 2017; Wilhelm et al. 1992).

There may be severe anemia caused by the destruction of erythrocytes or haemo-dilution due to impaired osmoregulation across the gill epithelium as dissolved oxygen levels declined significantly as a result of the significant reduction in hemoglobin concentration, packed cells volume, red blood cells, white blood cells, and platelets (Musa et al. 2013). The reduction in hemoglobin concentration may be the result of an increased rate of red blood cell breakdown and/or reduced rate of red blood cell formation due to the plant extract (Kuhn et al. 2017). The changes observed in the values of haematological indices in these studies were concentration-dependent and may be attributed to the concentrations and the duration of exposure to the toxicant.

As part of screening for liver disease, serum aminotransferases, total proteins, and albumin are considered to be the most common liver function tests. Morcos et al. (2015) describe these tests as the most common liver function tests. These enzymes are released into the bloodstream as a result of hepatocellular damage. As a result of hepatic injuries, cellular infiltration, and alterations in liver cell membrane function, serum AST and ALT levels increase Morcos et al. (2015). As for albumin levels, they are related to hepatic cell functions (Rezaei-Moghadam et al. 2012). In the present results, the toxic effect of xylene, toluene, and benzene lead to an increase in liver function. These results indicate that benzene is more hepatotoxic than toluene and xylene. Similar results were reported by Morcos et al. (2015) who observed increase of AST and ALT activities in mice exposure to xylene. Also, Benzene, toluene, and xylene treated rats showed increased liver functions, as observed by Kumar and Singh (2003). Also, AI-Sahhaf and Sarhan (2016) who observed increase ALT and AST in the sera of albino rat exposed to toluene. The plasma transaminase level of toluene-treated rats increased according to Ayan et al. (2012). It was described by Tas et al. (2011) that toluene treatment of rats resulted in significant increases in serum ALT and AST, and a decrease in serum albumin, but not in any increase in serum ALP or total bilirubin. Dere and Ari (2009) who observed a significant increase in the AST ALP and ALT activities (p < 0.05) in rats’ benzene-treated in comparison to those of controls (p > 0.05). A rise in liver enzymes could be caused by benzene affecting the organelles in cells in tissues. By influencing the organelles of the cell, Dere and Ari (2009) expect that enzyme activity will be indirectly influenced. Benzene treatment of fish increases enzyme activity in serum primarily because liver cytosol leaks these enzymes into the bloodstream Dere and Ari (2009). Furthermore, Rahman et al. (2000) suggested that the increased plasma ALP activities might be due to an increased permeability of the plasma membranes or cellular necrosis, which indicated the animals were under stress. Similar results in other materials were conducted by Ismail and Mahboub (2016), Sayed and Hamed (2017); Abou Khalil et al. (2017) who observed increased in AST, ALT serum glucose and total protein when exposure *Clarias gariepinus to* into 4- nonylphenol. A number of mechanisms have been suggested as to why this increase occurs, including liver damage, leakage of these enzymes, and activation of glucogenolysis (Bhattacharya et al. 2008; Winkaler et al. 2007).

SOD and CAT are important antioxidant enzymes for detoxication against ROS attack (Ighodaro & Akinloye 2018). SOD catalyzes the formation of water and oxygen in the presence of superoxide anions, while CAT breaks down hydrogen peroxide (H2O2) to water and oxygen (Ran et al. 2007). In the present results, the toxic effect of xylene, toluene and benzene lead to an increase in all antioxidant enzymes. The antioxidant enzymes levels in the liver were higher in benzene treated fish in comparison to toluene and xylene treated fish. Saleh et al. (2007) has been observed an increase in MDA levels (p < 0.01) of albino rats exposed to 0.5 ml/kg body weight benzene but observed a decrease in the levels of SOD and catalase concentration than control group. Benzene administration to albino rats increased their MDA levels in the same way as reported in other studies (Ahmad et al. 1994; Pandya et al. 1990). Also, Kumar and Singh (2003) observed increase in level of MDA and catalase in benzene, toluene and xylene treated rat in comparison to control. It has been shown that benzene significantly increased serum levels of MDA when administered to rats, in agreement with El- Batsh et al. (2015). According to Ibrahim et al. (2011), serum MDA levels were elevated in rats that had been injected with benzene, and antioxidant activity was decreased in those rats that had been injected with benzene. The release of free radicals causes the peroxidation of membrane lipids, which ruptures the lysosomal membranes, releases lysosomal enzymes, causes necrosis of the cell, and destroys the parenchyma resulting in an increase in serum MDA levels (Mehendale 2012). Based on previous studies conducted after exposure to 4-NP, the present study confirms those findings (Abou Khalil et al. 2017; Sayed et al. 2017, Sayed and Soliman 2018) who observed increased in ALP, SOD, catalase, and TAC.

The current study showed that xylene, benzene and toluene induced marked increase in the apoptotic erythrocytes of *O. niloticus* compared to control. Similar results were conducted by Sayed and Soliman (2018) who observed increase in percentages of DNA fragmentation and the apoptotic when fish exposed to 4- nonylphenol. Based on previous studies, the present study confirms those findings (Mekkawy et al. 2011, Sayed and Hamed 2017, Sayed et al. 2016). According to Jubendradass et al. (2012), male rats exposed to various doses of 4-NP exhibited a significant increase in apoptosis. Also, Zheng et al., (2014) observed increase in apoptosis of fish when exposed to Nichel.

In the present study, serum AST, ALT, and ALP activity elevated, indicating apoptosis in erythrocytes and liver necrosis (Kaplan 1988). The increased ROS caused by organic solvents can also stimulate apoptosis, which impairs the mitochondrial respiratory chain and affects membrane permeability (Zhang et al. 2012). The effect of chemical environmental contaminants on the apoptosis pathway in fish cells has been well-known for several years (Selvaraj et al. 2013; Zhang et al. 2012, 2013). Histopathology is considered as one basic technique in aquatic toxicity. In addition to providing an indication of the effects of various pollutants on organisms, histopathology also provides a link between the overall health of the ecosystem and the health of its inhabitants (Bernet et al. 1999), thus the exposure of Nile tilapia to three chemicals resulted in various degrees of histopathological alterations in liver, kidney and gills leading to different levels of lesion distribution.

Gills are good indicators of the quality of the water, since they provide models for studying environmental impact (Mallatt 1985, McKim & Erickson 1991). It is clear from the results of the present study that several changes are in agreement with those observed by Alvarez-Munoz et al. (2009), who observed epidermal lifting, fusion, and stagnation of the gill lamellae and vessels of the gill. It has been reported that degeneration and necrosis of epithelial cells are present in both primary and secondary lamellae of the secondary lamellae, and epithelial cells are disrupted from those of the pillar cells in De Silva and Samayawardhena,(2002) study. The gill lamellae in *Poecilia reticulate* were shorter, fusion was observed, lamellae were destroyed, vacuolation increased, and lamellae were irregular. Kelley (2007) points out that fusion of the secondary lamellae could decrease the surface area of the secondary lamellae contacting the water, thereby impairing blood-water exchange.

For vertebrates in general and fish in particular, the liver is essential in xenobiotic metabolism and excretion (Bernet et al. 1999). The present results showed that exposing fish for 15 days to organic solvent (BTX) showed clear adverse pathological changes in liver including hepatocytes dissociation, vacuolar degeneration of hepatocytes and Steatosis. Congestion and necrosis of central vein were also recorded in all treated fish. Thrombosis, melanomacrophages aggregation and coagulative necrosis of hepatocytes. These results are in agreement with the results of Meydan et al, (2019) after 15 days of toluene injection in albino rats described, sinusoid dilation, hemorrhage, vacuolization and necrosis. Clearly, toluene exhibits hepatotoxic effects. Our results are also closely related to the results of Ayanda et al., (2015) that examine the effect of glyphosate and paraquat, on liver of *Clarias gariepinus* juveniles for eight weeks. Our findings are also similar to previous reports on the liver of *Oncorhynchus mykiss* after exposure to gold nanoparticles in hepatocytes degeneration (Farkas et al. 2010). We also observed liver damage in freshwater catfish that were exposed to an organophosphate pesticide, as shown by Persis and Kalaiarasi (2001). They showed cord disarray, hypertrophy, disintegration of hepatocytes, lymphocytic infiltration, sinusoidal blood congestion, and hemorrhage. Benzene has a high vapor pressure, resulting in fast volatilization and a high toxicity whereas xylene has a low vapor pressure that results in high concentrations remaining in the test media. As well as insulin resistance and energy depletion, vacuolar changes of hepatocytes are also associated with cell death (Laurén et al. 1990). As a result of an increase in blood volume in the capillaries, congestion affects blood circulation (Rejeki &Mulyana 2008) which may be responsible for cellular degeneration and hepatic necrosis (Mohamed 2008). Nayak et al. (1996) reported that many of the liver vacuolated hepatocytes are adaptively altered to resist further exposure more than hydropic degeneration.

The observed results are probably due to the importance of the kidney as the major function of the renal tubules is the divalent ions excretion. Heavy metals and pesticides affect these cells, so degeneration in renal tubules indicates renal toxicity (Chaudhuri et al. 1999). In our study Nile tilapia exposed to BTX for 15 days, suffered from renal dysfunctions in the form of Glomerular swelling with melanomacrophages aggregation, diffuse interstitial inflammatory cell infiltration**,** necrosis, vacuolar degeneration in epithelial cells lining renal tubules and Interstitial hemorrhages between tubules. like our results, Li et al, (2015b) cleared that Toluene exposure results in structural and functional impairment of various organs. Renal function impairment as a result of renal tubule in workers exposed to a mixture of toluene and xylene (Akgül et al. 2011). Our results are closely related to the results of Ali et al., (2015) who reported that renal tubular injury was observed to in a 22-year old woman- inhalated pure toluene. Mostafa-Hedeab et al. (2015) reported damage of proximal cell with development of renal failure in rat kidney after toluene exposure. Ortiz et al., (2003) showed tubular necrosis, desquamation, and vacuolization of tubular epithelial cells in kidney of fishes exposed to linden. Ortiz et al., (2003) and Roy and Bhattacharya (2006) also reported similar results in fish exposed to linden and arsenic, respectively. It is difficult to find the cellular mechanisms by which BTX cause nephrotoxicity and renal dysfunction.

In conclusion, results of the present study clear the effects of BTX on Nile tilapia as fish model for environmental pollution studies. The gill, liver, and kidney damages were dependent on the type of the pollutant. When comparing histopathological changes in the BTX, xylene shows more damage than toluene while benzene was the least toxic. The findings suggest that hematological, biochemical, mutagenic, and histopathological changes are important biomarkers of environmental pollution studies. However, measurement of its bioaccumulation is essential for good understanding of its deleterious effects.

## Data Availability

All data generated or analyzed during this study are included in the research article.
